# Polydopamine-Functionalized CA-(PCL-*ran*-PLA) Nanoparticles for Target Delivery of Docetaxel and Chemo-photothermal Therapy of Breast Cancer

**DOI:** 10.3389/fphar.2018.00125

**Published:** 2018-02-21

**Authors:** Na Kong, Mei Deng, Xiu-Na Sun, Yi-Ding Chen, Xin-Bing Sui

**Affiliations:** ^1^Sir Run Run Shaw Hospital, Zhejiang University School of Medicine, Hangzhou, China; ^2^Institute of Translational Medicine, Zhejiang University, Hangzhou, China; ^3^Department of Surgical Oncology, The Second Affiliated Hospital, Zhejiang University School of Medicine, Hangzhou, China; ^4^Department of Medical Oncology, Holistic Integrative Oncology Institutes and Holistic Integrative Cancer Center of Traditional Chinese and Western Medicine, The Affiliated Hospital of Hangzhou Normal University, College of Medicine, Hangzhou Normal University, Hangzhou, China; ^5^Department of Cancer Pharmacology, Holistic Integrative Pharmacy Institutes, College of Medicine, Hangzhou Normal University, Hangzhou, China; ^6^Key Laboratory of Elemene Class Anti-cancer Chinese Medicine of Zhejiang Province and Engineering Laboratory of Development and Application of Traditional Chinese Medicine from Zhejiang Province, Hangzhou Normal University, Hangzhou, China

**Keywords:** polydopamine coating, star-shaped copolymer, aptamer, target delivery, chemo-photothermal therapy

## Abstract

Current limitations of cancer therapy include the lack of effective strategy for target delivery of chemotherapeutic drugs, and the difficulty of achieving significant efficacy by single treatment. Herein, we reported a synergistic chemo-photothermal strategy based on aptamer (Apt)-polydopamine (pD) functionalized CA-(PCL-*ran*-PLA) nanoparticles (NPs) for effective delivery of docetaxel (DTX) and enhanced therapeutic effect. The developed DTX-loaded Apt-pD-CA-(PCL-*ran*-PLA) NPs achieved promising advantages, such as (i) improved drug loading content (LC) and encapsulation efficiency (EE) initiated by star-shaped copolymer CA-(PCL-*ran*-PLA); (ii) effective target delivery of drugs to tumor sites by incorporating AS1411 aptamers; (iii) significant therapeutic efficacy caused by synergistic chemo-photothermal treatment. In addition, the pD coating strategy with simple procedures could address the contradiction between targeting modification and maintaining formerly excellent bio-properties. Therefore, with excellent bio-properties and simple preparation procedures, the DTX-loaded Apt-pD-CA-(PCL-*ran*-PLA) NPs effectively increased the local drug concentration in tumor sites, minimized side effects, and significantly eliminated tumors, indicating the promising application of these NPs for cancer therapy.

## Introduction

Breast cancer, the leading type of cancer for deaths among women worldwide, has caused over an estimated 2,421,698 deaths for the year 2015 (Global Burden of Disease Cancer Collaboration, 2017). Current approaches for breast cancer therapy are still limited by the level of our medical technology. For example, chemotherapy, which is one of the most common clinical methods for breast cancer therapy, is non-specific and always accompanied with serious side effects because of lacking effective carriers (Tao et al., 2013, 2015a; Shi J. et al., 2017; Zeng et al., 2017). Nano-biotechnology has shown promising prospects for changing the landscape of pharmaceutical and biomedical industries fundamentally (Shi et al., 2010; Tao et al., 2015b, 2017b,d; He et al., 2016; Wang et al., 2016, 2017; Hofmann et al., 2017; Niu et al., 2017; Qi et al., 2017; Shi B. et al., 2017; Shi J. et al., 2017; Xu et al., 2017a,b). The key to solve the side effects caused by cytotoxic anticancer drugs is to increase the local effective drug concentrations in the tumor sites, which could be addressed by employing polymeric nanoparticles (NPs) as highly promising target drug delivery systems (Kamaly et al., 2016; Kakkar et al., 2017; Nie et al., 2017; Tao et al., 2017c; Xu et al., 2017). In addition, due to the molecular complexity of cancers, treatment based on monotherapies is suboptimal and can not effective eliminated tumors (Greco and Vicent, 2009; Sun et al., 2011). By combining two or more therapies with different therapeutic mechanisms in one nano-system (e.g., chemotherapy and photothermal therapy), a promising strategy could be achieved to significantly enhance the therapeutic efficacy and have a better long-term prognosis (Cheng et al., 2017; Tao et al., 2017d). Thus, developing multifunctional target delivery platforms that intelligently integrated with different therapeutic approaches is urgently needed for effective cancer therapy.

Aptamers, which are essentially single-stranded DNA or RNA oligonucleotides and have specific 3D structures, are able to bind various biological targets on cancer cells with unique specificity and high affinity (Farokhzad et al., 2004; Dhar et al., 2008; Xu et al., 2016). Aptamers possess distinct advantages over antibodies. Examples include easier production without the use of animal, smaller size, readily availability, lower molecular weights, non-immunogenicity, higher targeting efficacy and *in vivo* stability (Jiang et al., 1996; Sun et al., 2014). Therefore, aptamers have been widely used as effective targeting agents for cancer therapy and diagnosis (Zhu D. et al., 2016). As one of the most commonly used DNA aptamers, AS1411 is able to effectively target a wide variety of cancer cells including breast cancer cells (MCF-7) (Keefe et al., 2010). Because of the excellent performance on the targeting efficacy, AS1411 can be incorporated in the surface modification of NPs as an effective targeting ligand for breast cancer therapy. Nevertheless, major concerns still exist for NPs prepared by pre-functionalized polymers. For example, the synthesis of AS1411 aptamer-polymer conjugates could be inefficient, possibly altering the chemical properties, lengthy with high cost, and at a risk of compromising the ability for drug encapsulation of polymers (Ho and Leclerc, 2004). Another method to conjugate AS1411 on the surface of NPs is surface modification of prepared NPs, but this will be cumbersome for NPs lacking reactive functional groups, which still require reacting with reactive linkers or coupling agents followed by exhaustive purification processes in order to remove excess reactants and catalysts (Takahashi et al., 2010).

In order to address these issues, a simple and versatile surface modification strategy based on polydopamine (pD) was adopted in this study. As reported by Park et al., surface modification with pD is applicable to various NP-based drug carriers no matter what the types of ligands (e.g., small molecules, peptides, or polymers) and how is the chemical reactivity of NPs (Park et al., 2014). Briefly, dopamine catechol could be oxidized to quinone followed by reactions with other quinones or catechols to form pD in weak alkaline conditions, gradually forming a water-insoluble polymeric film on NP surface during the process (Jiang et al., 2011; Chang et al., 2016). Afterward, ligands possessing amine or thiol groups could be simply conjugated on the surface of pD-coated NPs via Michael addition or Schiff base reactions (Lee et al., 2007, 2009; Tao et al., 2016). Besides the function of surface modification, pD and its derivatives have been widely reported as effective photothermal agents, which could contribute to phtotothermal therapy of breast cancer in the meantime. As phtotothermal therapy is a potent technique for cancer therapy because of its high selectivity and minimal invasiveness (Huang et al., 2013; Miao et al., 2016; Song et al., 2016; Tao et al., 2017a), it will be rather promising to initiate a synergistic target chemo-photothermal strategy (Tao et al., 2017d).

It has been reported that star-shaped copolymers possess many unique advantages for developing NP-based drug delivery platforms (Mei et al., 2013). To build the core of the NPs, a star-shaped co-polymer cholic acid functionalized poly(𝜀-caprolactone-*ran*-lactide) [CA-(PCL-*ran*-PLA)] was successfully synthesized according to a previous report (Tao et al., 2014), which has been demonstrated with many advantages such as improved bioavailability (i.e., solubility, stability, and permeability) of anticancer drugs, increased drug loading content (LC) and encapsulation efficiency (EE), controlled drug release profiles, and excellent antitumor efficacy. Herein, we used a nano-precipitation method to prepare CA-(PCL-*ran*-PLA NPs, coated the developed NPs with pD layer [pD-CA-(PCL-*ran*-PLA) NPs], and target functionalizing the pD-coated NPs with AS1411 aptamers [Apt-pD-CA-(PCL-*ran*-PLA) NPs]. The newly developed NPs were characterized by surface morphology, LC and EE, stability, photothermal properties and drug release profiles. The *in vitro* and *in vivo* targeting effect of these NPs were also accessed. With excellent biocompatibility, the docetaxel (DTX)-loaded Apt-pD-CA-(PCL-*ran*-PLA) NPs were demonstrated with a significant antitumor efficacy through a target chemo-photothermal strategy.

## Materials and Methods

### Materials

Colic acid, D,L-Lactide (3,6-dimethyl-1,4-dioxane-2,5-dione, C_6_H_8_O_4_), 4-(dimethylamino)pyridine (DMAP), 1,3-diisopropylcarbodiimide (DCC), 2-(3,4-dihydroxyphenyl) ethylamine (dopamine) hydrochloride, 3-(4,5-dimethylthiazol-2-yl)-2,5-diphenyltetrazolium bromide (MTT), and Stannous octoate [Sn(Oct)_2_] were purchased from Sigma (St. Louis, MO, United States). 𝜀-caprolactone (CL) was purchased from Acros Organics (Geel, Belgium). Commercial Taxotere^®^ and docetaxel (DTX) were provided by Shanghai Jinhe Bio-tech Co., Ltd (Shanghai, China). Methanol and acetonitrile were provided by EM Science (ChromAR, HPLC grade, Mallinckrodt Baker, United States). All other agents used were of analytical reagent grade. Boon Environmental Tech. Industry Co., Ltd (Tianjin, China) provided the ultrahigh pure water utilized throughout all the studies. Human breast cancer cell line MCF-7 was purchased from American Type Culture Collection (ATCC, Rockville, MD, United States).

### Synthesis of DTX/Apt-pD-CA-(PCL-*ran*-PLA) NPs

#### Preparation of DTX/CA-(PCL-*ran*-PLA) NPs

The star-shaped random copolymer CA-(PCL-*ran*-PLA) was synthesized through the ring opening copolymerization, and provided by Prof. Lin Mei’s group at Tsinghua University (Tao et al., 2014). We adopted a modified nanoprecipitation method with water/acetone system to develop DTX-loaded CA-(PCL-*ran*-PLA) NPs (DTX/CA-(PCL-*ran*-PLA) NPs) (Zeng et al., 2013a, 2015a; Zhang et al., 2014a,b, 2015; Wang et al., 2015; Wu et al., 2015). In brief, 200 mg of CA-(PCL-*ran*-PLA) copolymers and 20 mg of DTX were dissolved in 16 mL of acetone. The solution was then titrated into 200 mL of 0.03% (w/v) TPGS aqueous solution with continuous stirring. After stirring overnight at a speed of 800 rpm to remove acetone, the pure DTX/CA-(PCL-*ran*-PLA) NPs were obtained by centrifugation at a speed of 20,000 rpm (4°C) for 20 min, followed by three times washing in 20 mL of deionized water to remove TPGS emulsifier and unencapsulated DTX. Finally, pre-calculated amount of the obtained DTX/CA-(PCL-*ran*-PLA) NPs were weighted for pD coating, while the left NPs were dispersed in deionized (DI) water and lyophilized 2 days for further use.

#### Surface Modification with pD Layers

The DTX/pD-CA-(PCL-*ran*-PLA) NPs were prepared by incubating DTX/CA-(PCL-*ran*-PLA) NPs in 0.1 mg/mL dopamine hydrochloride/10 mM Tris buffer solution (pH 8.5) under magnetic stirring at room temperature. After 6 h of reaction, the suspensions gradually turned darker, indicating that dopamine was successfully polymerized. The obtained DTX/pD-CA-(PCL-*ran*-PLA) NPs were centrifuged (12,000 rpm, 30 min) and lyophilized for conjugation of targeting AS1411 aptamers.

#### Conjugation of Targeting AS1411 Aptamers

The sequence of AS1411 aptamers used in this study contains 10 extra T bases at the 3-terminus (5′-GGT GGT GGT GGT TGT GGT GGT GGT GGT TTT TTT TTT-thiol-3′). The SH-terminated aptamers were conjugated on the surface of pD-coated NPs through a Michael addition reaction (Park et al., 2014). Briefly, the DTX/pD-CA-(PCL-*ran*-PLA) NPs were dissolved in the Tris buffer (pH 8.0) containing aptamers with final concentrations of NPs and AS1411 aptamers at 1 and 0.5 mg/mL, respectively. After 2 h of magnetic stirring at room temperature, the resulting DTX/Apt-pD-CA-(PCL-*ran*-PLA) NPs were centrifuged (12,000 rpm, 30 min), washed three times with deionized water and lyophilized 2 days for further use.

All the fluorescent coumarin-6 (C6)-loaded NPs (i.e., C6/CA-(PCL-*ran*-PLA) NPs, C6/pD-CA-(PCL-*ran*-PLA) NPs, and C6/Apt-pD-CA-(PCL-*ran*-PLA) NPs) were prepared with the same method at each of the 3 steps described above.

### Characterization of DTX/Apt-pD-CA-(PCL-*ran*-PLA) NPs

The size of DTX/CA-(PCL-*ran*-PLA) NPs, DTX/pD-CA-(PCL-*ran*-PLA) NPs, and DTX/Apt-pD-CA-(PCL-*ran*-PLA) NPs were performed by Malvern Mastersizer 2000 (Zetasizer Nano ZS90, Malvern Instruments Ltd., United Kingdom). The transmission electron microscopy (TEM, Tecnai G2 F30, FEI Company, Hillsboro, OR, United States) was used to access the surface morphology of these NPs. The photothermal properties of these NPs were determined by recording the temperature changes of various solutions with different concentrations of NPs (62.5, 125, 250, and 500 μg/mL) under the irradiation of an 808 nm NIR laser (Shanxi Kaisite Electronic Technology Co., Ltd., Xi’an, China) at a power density of 1.0 W/cm^2^. To study the effect of power density on the photothermal effect of DTX/Apt-pD-CA-(PCL-*ran*-PLA) NPs, NP solution at the concentration of 500 μg/mL was irradiated by different power densities (0.5–2.0 W/cm^2^) and the changes of temperature were recorded. The temperatures were monitored by an infrared thermal imaging camera (TI100 Infrared Camera FLK-TI100 9HZ, FLUKE).

### Drug Loading Content (LC) and Encapsulation Efficiency (EE)

The LC and EE of the DTX/CA-(PCL-*ran*-PLA) NPs, DTX/pD-CA-(PCL-*ran*-PLA) NPs, and DTX/Apt-pD-CA-(PCL-*ran*-PLA) NPs were determined by HPLC (LC 1200, Agilent Technologies, Santa Clara, CA, United States). In brief, 5 mg of NPs were dissolved in 1 mL of DCM under vigorous vortexing, and the prepared solution was transferred to 5 mL of mobile phase containing acetonitrile and DI water (50:50, v/v). In order to get a clear solution for HPLC, a nitrogen stream was used to evaporate DCM for 15 min. A reverse-phase C-18 column (150 × 4.6 mm, 5 μm, C18, Agilent Technologies, Santa Clara, CA, United States) was utilized at 35°C, and the flow rate of mobile phase was set at 1.0 mL/min. A UV/VIS detector was used to detect the column effluent at 227 nm. The drug LC and EE of these NPs were calculated by the following equations respectively (*n* = 3).

$$\begin{array}{c} \mathrm{LC}(\%)=\frac{\mathrm{Weight of DTX in NPs}}{\mathrm{Weight of NPs}}\times 100\%\\ \mathrm{EE}(\%)=\frac{\mathrm{Weight of DTX in NPs}}{\mathrm{Weight of the feeding DTX}}\times 100\% \end{array}$$

### *In Vitro* Drug Release Profiles

In order to study the *in vitro* DTX release profiles, 5 mg of the lyophilized NPs were dispersed in 5 mL of PBS (pH 7.4, containing 0.1% w/v Tween 80). Tween 80 was used to increase the solubility of DTX while avoid the binding of DTX on the tube wall. Afterward, the NP suspension was transferred into a dialysis membrane bag (MWCO = 3,500, Shanghai Sangon, China) which immersed in 15 mL of fresh PBS in a centrifuge tube. The whole tube was then transferred into an orbital water bath and shaken at a speed of 120 rpm (37°C). At designated time points, 10 mL of release medium was picked out for HPLC analysis. After changing 15 mL of fresh PBS solution, the tuber was transferred back to the shaker for continuous tests. The cumulative release of DTX from the DTX/CA-(PCL-*ran*-PLA) NPs, DTX/pD-CA-(PCL-*ran*-PLA) NPs, and DTX/Apt-pD-CA-(PCL-*ran*-PLA) NPs was plotted against time.

### Cellular Uptake of Fluorescent NPs

MCF-7 cells were cultured in Dulbecco’s Modified Eagle’s Medium (DMEM) supplemented with 10% fetal bovine serum (FBS), 100 mg/mL streptomycin, and 100 U/mL penicillin in 5% CO_2_ at 37°C. The cell culture was stayed in 95% air humidified atmosphere. The cells were incubated with 250 μg/mL C6/CA-(PCL-*ran*-PLA) NPs, C6/pD-CA-(PCL-*ran*-PLA) NPs, or C6/Apt-pD-CA-(PCL-*ran*-PLA) NPs for 2 h, washed with cold PBS three times, and fixed by cold methanol for 20 min. After that, the nuclei were counterstained with DAPI for 10 min and washed twice with PBS. In order to visualize the cells, the chambers were mounted onto the confocal laser scanning microscope (CLSM, Olympus Fluoview FV-1000, Tokyo, Japan) with a blue channel excited at 340 nm and a green channel excited at 485 nm.

For quantitative analysis, MCF-7 cells were plated in 96-well black plates and incubated overnight at its initial density of 1 × 10^4^ cells/well. Then the cells were equilibrated with Hank’s buffered salt solution (HBSS) for 1 h at 37°C, and C6/CA-(PCL-*ran*-PLA) NPs, C6/pD-CA-(PCL-*ran*-PLA) NPs, or C6/Apt-pD-CA-(PCL-*ran*-PLA) NPs were added at concentrations of 100, 250, and 500 μg/mL, respectively. After 2 h of incubation, the medium was removed and the wells were washed three times with 50 μL of cold PBS. Finally, 50 μL of 0.5% Triton X-100 in 0.2 N sodium hydroxide was added to each sample to lyse the MCF-7 cells.

For flow cytometric (FCM) experiments, MCF-7 cells were seeded in 6-well plates (at a density of 1 × 10^5^ cells/well), and treated with 500 μg/mL of C6/CA-(PCL-*ran*-PLA) NPs, C6/pD-CA-(PCL-*ran*-PLA) NPs, or C6/Apt-pD-CA-(PCL-*ran*-PLA) NPs for 1 h at 37°C, respectively. After removing the cell culture medium, the cells were washed with PBS twice, digested by trypsin, and harvested by centrifugation. Finally, the fluorescence intensity of C6 was detected by a flow cytometer (BD Biosciences, San Jose, CA, United States) at an excitation wavelength of 488 nm and an emission wavelength of 530 nm.

### *In Vitro* Cell Viability Studies

In order to evaluate the biocompatibility of drug-free NPs and the *in vitro* antitumor efficacy of the DTX-loaded NPs, MCF-7 cells were seeded in 96-well plates (at a density of 5,000 cells/well) and incubated overnight.

For the biocompatibility studies, the MCF-7 cells were incubated with drug-free CA-(PCL-*ran*-PLA) NPs, pD-CA-(PCL-*ran*-PLA) NPs, or Apt-pD-CA-(PCL-*ran*-PLA) NPs at different NP concentrations (25, 125, 250, and 500 μg/mL) and incubation time (24 and 48 h). Afterward, the previous mediums were changed with MTT-contained DMEM (5 mg/mL) and the cells were incubated for an additional 4 h. Then MTT was removed and DMSO was added to dissolve the formazan crystals (2 h, dark, 37°C). The absorbance at 570 nm was measured using a microplate reader (Bio-Rad Model 680, United Kingdom). Control group with untreated cells was used as 100% of viability, and cells incubated with MTT-free medium were utilized as blank to calibrate the spectrophotometer to zero absorbance.

For the *in vitro* antitumor efficacy studies, (i) Chemotherapy groups: the MCF-7 cells were incubated with commercial Taxotere^®^, DTX/CA-(PCL-*ran*-PLA) NPs, DTX/pD-CA-(PCL-*ran*-PLA) NPs, or DTX/Apt-pD-CA-(PCL-*ran*-PLA) NPs at 0.25, 2.5, 12.5, and 25 μg/mL equivalent DTX concentrations for 24 h and 48 h, respectively. (ii) Irradiation groups: DTX/CA-(PCL-*ran*-PLA) NPs, DTX/pD-CA-(PCL-*ran*-PLA) NPs, or DTX/Apt-pD-CA-(PCL-*ran*-PLA) NPs at 0.25, 2.5, 12.5, and 25 μg/mL equivalent DTX concentrations were added into the medium of MCF-7 cells and immediately irradiated with an 808 nm NIR laser for 10 min at a power density of 1.5 W/cm^2^. Afterward, MCF-7 cells from all the groups were further cultured for 24 and 48 h, respectively. Untreated cells only received NIR laser irradiation were used as controls. The same MTT method was used to test the cell viability of cells from all these groups in order to access the *in vitro* antitumor efficacy.

### Xenograft Breast Tumor Model

The 4–5 weeks old of female severe combined immunodeficient (SCID) nude mice were purchased from the Institute of Laboratory Animal Sciences, Chinese Academy of Medical Science. The Administrative Committee on Animal Research in Zhejiang University approved the protocols for animal studies. A total volume of 200 μL MCF-7 cells in PBS were implanted subcutaneously on the backs of mice at a dosage of 2 × 10^6^ cells per mouse. The tumor growth in each mouse was observed at frequent intervals. The tumor size was measured by a vernier caliper, and tumor volume (V) was calculated by this formulation: 4π/3 × (length/2) × (width/2)^2^. About 95% of the mice developed a tumor with an average volume of about 100 mm^3^ after 2 weeks.

### *In Vivo* Photothermal Imaging and *in Vivo* Antitumor Efficacy

The photothermal imaging studies were performed when tumor size reached about 400 mm^3^. The mice were intravenously (i.v.) injected with Saline, DTX/pD-CA-(PCL-*ran*-PLA) NPs, and DTX/Apt-pD-CA-(PCL-*ran*-PLA) NPs at the same volume and dose. After 12 h of injection, NIR laser irradiation was performed (808 nm, 1.5 W/cm^2^, 10 min) at tumor sites. The temperature changes of tumor sites, as well as infrared thermographic maps, were recorded by an infrared thermal imaging camera (TI100 Infrared Camera FLK-TI100 9HZ, FLUKE).

For *in vivo* antitumor efficacy studies, Apt-pD-CA-(PCL-*ran*-PLA) NPs were chosen based on their excellent performance on *in vitro* cellular targeting, *in vitro* antitumor efficacy, and *in vivo* tumor targeting. Different treatments were performed when the tumor volume reached approximately 100 mm^3^. The tumor-bearing mice were randomly divided into five groups (*n* = 5), and different treatments were performed on Day 0, 4, 8, and 12: (1) Saline, (2) Taxotere^®^, (3) DTX/Apt-pD-CA-(PCL-*ran*-PLA) NPs, (4) Apt-pD-CA-(PCL-*ran*-PLA) NPs + NIR, and (5) DTX/Apt-pD-CA-(PCL-*ran*-PLA) NPs + NIR. All the formulations were i.v. injected. Taxotere^®^ and DTX/Apt-pD-CA-(PCL-*ran*-PLA) NPs were injected at a same DTX dose of 10 mg/kg in 100 μL PBS. Apt-pD-CA-(PCL-*ran*-PLA) NPs were injected at a same NP dose with DTX/Apt-pD-CA-(PCL-*ran*-PLA) NPs. NIR laser irradiation was performed (808 nm, 10 min, 1.5 W/cm^2^) after 12 h injection of NPs. The tumor size and body weights were recorded every 2 days. After 14 days of treatment, the mice were humanely executed. Tumor growth profiles were recorded to evaluate the antitumor efficacy in this study.

Another batch of SCID mice bearing MCF-7 tumor xenograft were further used to access the antitumor efficacy of the mice through recording their survival time after receiving the same treatments mentioned above. The mice with similar physical status (i.e., age, body weight, and 100 mm^3^ of tumor volume) were randomly divided into five groups (*n* = 5). The survival results were presented as Kaplane–Meier plots and evaluated using a log-rank test.

### Statistical Methodology

All tests were performed at least three times in all studies unless otherwise stated. The results are expressed as mean ± SD, and the statistical significance of the results was determined by the Student’s *t*-test. The results were considered to be statistically significant if *P* < 0.05.

## Results and Discussion

### Preparation of DTX/Apt-pD-CA-(PCL-*ran*-PLA) NPs

**FIGURE 1:**
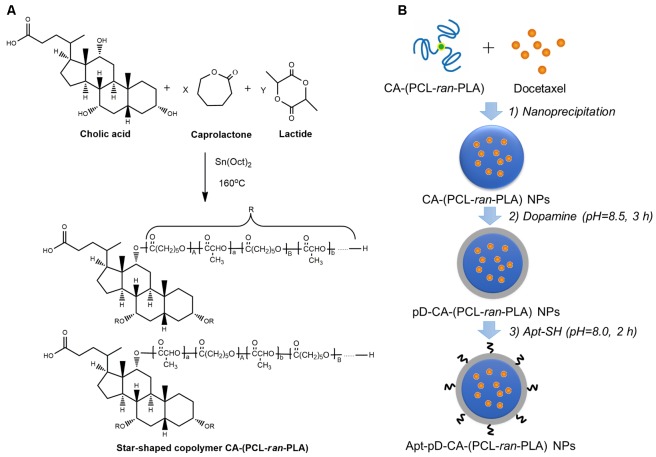
Illustration for the synthesis of **(A)** star-shaped CA-(PCL-*ran*-PLA) copolymer, and **(B)** DTX/Apt-pD-CA-(PCL-*ran*-PLA) NPs.

The star-shaped random copolymer CA-(PCL-*ran*-PLA) was synthesized through a the ring opening copolymerization of 𝜀-caprolactone (CL) and D,L-lactide (LA) with colic acid (CA) as an initiator (**Figure 1A**) (Tao et al., 2014). The fabrication of DTX-loaded Apt-pD-CA-(PCL-*ran*-PLA) NPs (DTX/Apt-pD-CA-(PCL-*ran*-PLA) NPs) was illustrated in **Figure 1B**: (i) First, a modified nano-precipitation method, which is a mild and effective way to prepare drug-loaded NPs (Zeng et al., 2013a, 2015b), was applied to prepare DTX/CA-(PCL-*ran*-PLA) NPs. (ii) Second, the prime-coating of pD layer was achieved through an oxidative polymerization reaction (pH 8.5) to prepare DTX/pD-CA-(PCL-*ran*-PLA) NPs. (iii) Finally, conjugation of aptamers on the surface of DTX/pD-CA-(PCL-*ran*-PLA) NPs could be fulfill via a Michael addition reaction in a weak alkaline solution (pH 8.0). The suspensions gradually turned darker in the second step after dopamine hydrochloride was added, indicating the formation of pD layer. A 26-mer SH-terminated DNA aptamer-AS1411, which possesses a thiol group at the 3′- end for effective conjugation to the pD layer, was applied to provide high affinity to interact with breast cancer cells (Cao et al., 2009).

### Characterization of DTX/Apt-pD-CA-(PCL-*ran*-PLA) NPs

**FIGURE 2:**
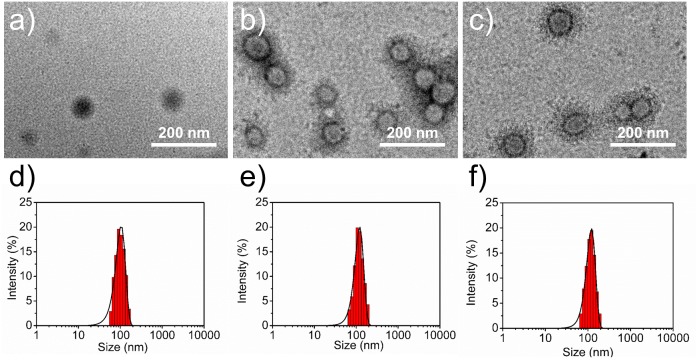
Surface morphology (TEM images) of **(a)** DTX/CA-(PCL-*ran*-PLA) NPs, **(b)** DTX/pD-CA-(PCL-*ran*-PLA) NPs, and **(c)** DTX/Apt-pD-CA-(PCL-*ran*-PLA) NPs. Size distribution of **(d)** DTX/CA-(PCL-*ran*-PLA) NPs, **(e)** DTX/pD-CA-(PCL-*ran*-PLA) NPs, and **(f)** DTX/Apt-pD-CA-(PCL-*ran*-PLA) NPs.

**FIGURE 3:**
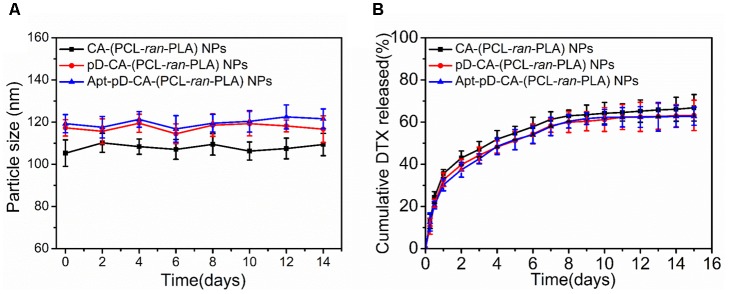
**(A)** Particle stability of DTX/CA-(PCL-*ran*-PLA) NPs, DTX/pD-CA-(PCL-*ran*-PLA) NPs, and DTX/Apt-pD-CA-(PCL-*ran*-PLA) NPs in PBS, respectively. **(B)** *In vitro* drug release profiles of DTX/CA-(PCL-*ran*-PLA) NPs, DTX/pD-CA-(PCL-*ran*-PLA) NPs, and DTX/Apt-pD-CA-(PCL-*ran*-PLA) NPs.

The size and surface properties of polymeric NPs, which play a vital role on their drug release, endocytosis and *in vivo* pharmacokinetics (Tao et al., 2016; Behzadi et al., 2017; Ding et al., 2017), have been well-characterized in this study. As shown in **Figures 2a–c**, the surface morphology of DTX/CA-(PCL-*ran*-PLA) NPs, DTX/pD-CA-(PCL-*ran*-PLA) NPs, and DTX/Apt-pD-CA-(PCL-*ran*-PLA) NPs was characterized through TEM images. It could be obviously observed that spherical films have been deposited on the surface of DTX/pD-CA-(PCL-*ran*-PLA) NPs and DTX/Apt-pD-CA-(PCL-*ran*-PLA) NPs, which is quite different from bare DTX/CA-(PCL-*ran*-PLA) NPs and gives direct evidences on the formation of pD layers. The average size distribution in TEM images is about 80 nm for DTX/CA-(PCL-*ran*-PLA) NPs, and about 95 nm for DTX/pD-CA-(PCL-*ran*-PLA) NPs and DTX/Apt-pD-CA-(PCL-*ran*-PLA) NPs (**Figures 2d–f**). As presented in **Table 1**, the average DLS size of DTX/CA-(PCL-*ran*-PLA) NPs was 103.4 ± 3.3 nm (PDI 0.126), while that of DTX/pD-CA-(PCL-*ran*-PLA) NPs was 120.3 ± 4.6 nm (PDI 0.115) and that of DTX/Apt-pD-CA-(PCL-*ran*-PLA) NPs was 124.6 ± 5.1 nm (PDI 0.123). The increased DLS size of DTX/pD-CA-(PCL-*ran*-PLA) NPs and DTX/Apt-pD-CA-(PCL-*ran*-PLA) NPs could be attributed to the thickness of pD layers formed on the surface of DTX/CA-(PCL-*ran*-PLA) NPs, which also indicates the successful modification of pD layers through the oxidative polymerization reaction. With the mean hydrodynamic size of NPs ranging from about 100–120 nm in diameter, they are perfectly fit for high accumulation in tumor vasculature driven by the influence of the enhanced permeability and retention (EPR) effect (Zhu et al., 2015; Zhu X. et al., 2016, 2017; Liu et al., 2017). The smaller size obtained from TEM images (compared with DLS testing) may be contributed to the shrink and collapse of NPs when in the dry state (Tao et al., 2013). The stability of NPs was accessed by monitoring particle size in PBS over a span of 2 weeks (**Figure 3A**). The average size of all the NPs hardly changed during the testing period, indicating a great stability of the NPs.

**Table 1:** Characterization of DTX/CA-(PCL-*ran*-PLA) NPs, DTX/pD-CA-(PCL-*ran*-PLA) NPs and DTX/Apt-pD-CA-(PCL-*ran*-PLA) NPs.

Samples (*n* = 3)	Size (nm)	PDI	ZP (mV)	LC (%)	EE (%)
DTX/CA-(PCL-*ran*-PLA) NPs	103.4 ± 3.3	0.126	-17.8 ± 3.9	10.02 ± 0.28	95.01 ± 2.16
DTX/pD-CA-(PCL-*ran*-PLA) NPs	120.3 ± 4.6	0.115	-18.6 ± 3.6	9.98 ± 0.39	94.31 ± 1.98
DTX/Apt-pD-CA-(PCL-*ran*-PLA) NPs	124.6 ± 5.1	0.123	-19.2 ± 5.2	9.73 ± 0.46	94.18 ± 2.76

*PDI, polydispersity index; ZP, zeta potential; LC, loading content; EE, encapsulation efficiency, *n* = 3.*

As shown in **Table 1**, the absolute value of zeta potential of DTX/Apt-pD-CA-(PCL-*ran*-PLA) NPs (-19.2 ± 5.2 mV) increased slightly or kept similarly compared to DTX/pD-CA-(PCL-*ran*-PLA) NPs (-18.6 ± 3.6 mV) and DTX/CA-(PCL-*ran*-PLA) NPs (-17.8 ± 3.9 mV). The negative charge of NPs could benefit the blood compatibility and passive accumulation of the NPs in tumor sites through reducing clearance by the reticuloendothelial system (RES) including liver. Moreover, **Table 1** also showed that the LC and EE of DTX/Apt-pD-CA-(PCL-*ran*-PLA) NPs (9.73 ± 0.46%, 94.18 ± 2.76%) were almost the same as those of DTX/pD-CA-(PCL-*ran*-PLA) NPs (9.98 ± 0.39%, 94.31 ± 1.98%) and DTX/CA-(PCL-*ran*-PLA) NPs (10.02 ± 0.28%, 95.01 ± 2.16%), indicating this strategy is facile and does not affect the effectiveness of drug LC and EE.

### *In Vitro* Drug Release Kinetics

**Figure 3B** showed the *in vitro* DTX release from DTX/CA-(PCL-*ran*-PLA) NPs, DTX/pD-CA-(PCL-*ran*-PLA) NPs, and DTX/Apt-pD-CA-(PCL-*ran*-PLA) NPs in the release medium (pH 7.4, PBS containing 0.1% w/v Tween 80) at 37°C. An initial burst release of DTX could be observed within the first 48 h, as about 40% of the loaded drugs were released in all kinds of NPs. At the end of our release studies (Day 15), about 66.8, 63.2, and 62.8% of DTX were released from DTX/CA-(PCL-*ran*-PLA) NPs, DTX/pD-CA-(PCL-*ran*-PLA) NPs, and DTX/Apt-pD-CA-(PCL-*ran*-PLA) NPs, respectively. Notably, the release rates from DTX/CA-(PCL-*ran*-PLA) NPs, DTX/pD-CA-(PCL-*ran*-PLA) NPs, and DTX/Apt-pD-CA-(PCL-*ran*-PLA) NPs were quite similar, which means the pD modification and AS1411 functionalization did not actually change the drug release properties of the prepared NPs. Therefore, Apt-pD-CA-(PCL-*ran*-PLA) NPs may be a promising drug delivery platform in nano-biotechnology and nanomedicines.

### *In Vitro* Photothermal Properties of DTX/Apt-pD-CA-(PCL-*ran*-PLA) NPs

**FIGURE 4:**
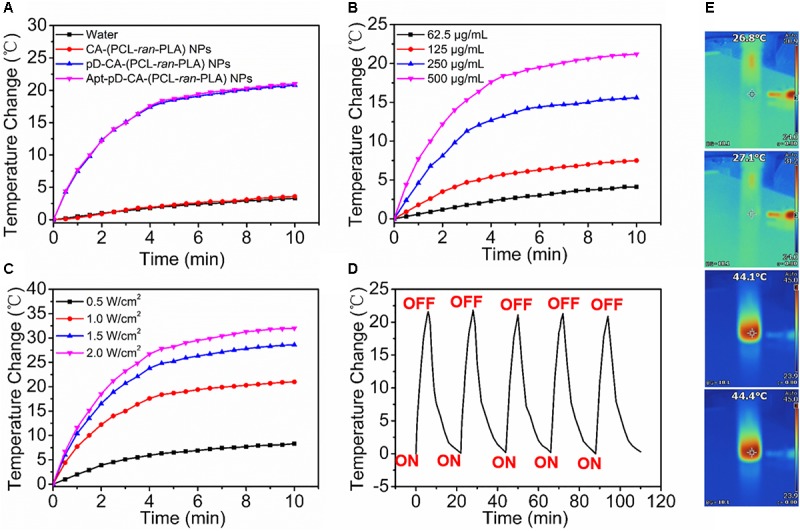
**(A)** Photothermal heating curves of DI water, DTX/CA-(PCL-*ran*-PLA) NPs, DTX/pD-CA-(PCL-*ran*-PLA) NPs, and DTX/Apt-pD-CA-(PCL-*ran*-PLA) NPs solution under an 808 nm laser irradiation (1.0 W/cm^2^) for 10 min. **(B)** Photothermal heating curves of the DTX/Apt-pD-CA-(PCL-*ran*-PLA) NPs solution with different concentrations (62.5, 125, 250, and 500 μg/mL). **(C)** Photothermal heating curves of the DTX/Apt-pD-CA-(PCL-*ran*-PLA) NPs solution with different power intensities of irradiation (0.5, 1.0, 1.5, and 2.0 W/cm^2^). **(D)** Heating of a suspension of the DTX/Apt-pD-CA-(PCL-*ran*-PLA) NPs in water for five laser on/off cycles with an 808 nm NIR laser (1.0 W/cm^2^). **(E)** IR thermal image of DI water, DTX/CA-(PCL-*ran*-PLA) NPs, DTX/pD-CA-(PCL-*ran*-PLA) NPs, and DTX/Apt-pD-CA-(PCL-*ran*-PLA) NPs (from top to bottom) solution under continuous NIR laser irradiation (1.0 W/cm^2^) for 10 min.

The photothermal properties of DTX/Apt-pD-CA-(PCL-*ran*-PLA) NPs was accessed by testing the temperature changes under the irradiation of an 808-nm NIR laser. **Figures 4A,E** showed a rapid temperature increase of ∼20°C was achieved by DTX/pD-CA-(PCL-*ran*-PLA) NPs and DTX/Apt-pD-CA-(PCL-*ran*-PLA) NPs (500 μg/mL) after 10 min of irradiation with the NIR laser (1.0 W/cm^2^). However, DI water and the DTX/CA-(PCL-*ran*-PLA) NPs showed little temperature change at totally the same conditions, demonstrating the superior photothermal effect of these NPs was majorly attributed to the pD layers. As shown in **Figure 4B**, the photothermal effect of DTX/Apt-pD-CA-(PCL-*ran*-PLA) NPs had a concentration-dependent relationship. The solutions of DTX/Apt-pD-CA-(PCL-*ran*-PLA) NPs also exhibited a laser power intensity-dependent manner as the temperatures increased with the rise of laser power intensity (**Figure 4C**). Moreover, the temperature changes did not exhibit noticeable difference during five cycles of “laser on”-“laser off” (**Figure 4D**), indicating an excellent photo-stability of DTX/Apt-pD-CA-(PCL-*ran*-PLA) NPs. Therefore, DTX/Apt-pD-CA-(PCL-*ran*-PLA) NPs have a good photothermal effect and a superior photostability, which could be applied in cancer photothermal therapy.

### Endocytosis of Fluorescent NPs and *in Vitro* Cellular Targeting

**FIGURE 5:**
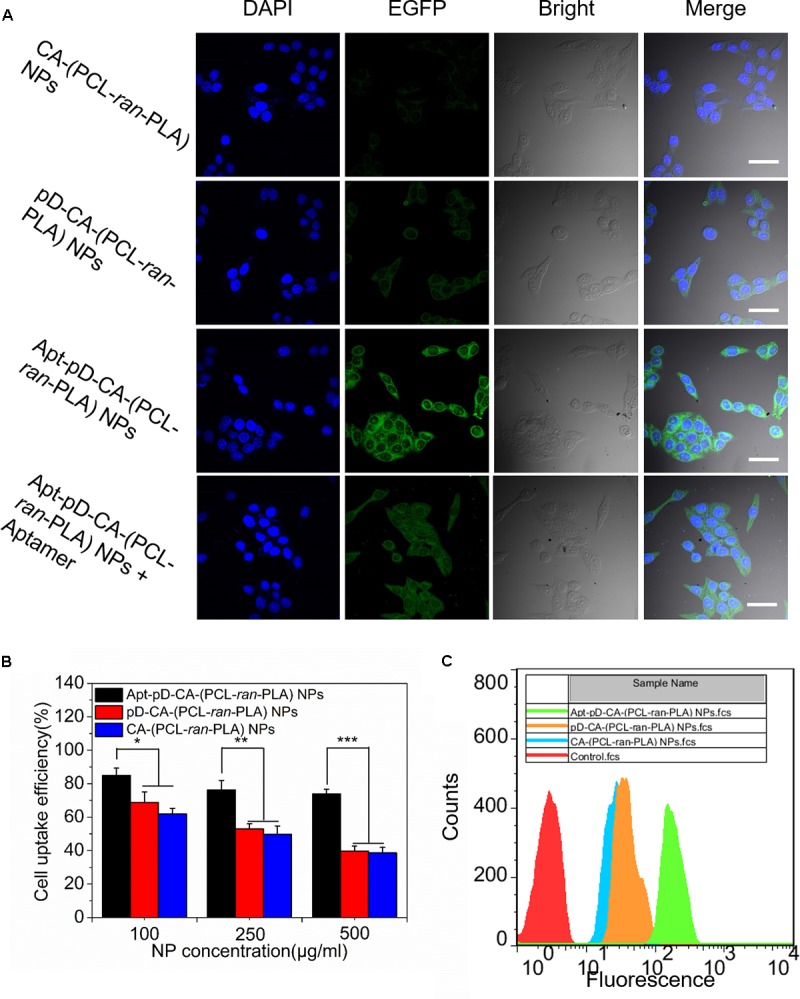
Cell uptake of coumarin 6-loaded CA-(PCL-*ran*-PLA) NPs, pD-CA-(PCL-*ran*-PLA) NPs and Apt-pD-CA-(PCL-*ran*-PLA) NPs. **(A)** CLSM images of MCF-7 cells after 2 h-incubation (scale bar: 40 μm). **(B)** Cellular uptake efficiency of different NPs in MCF-7 cells after 2 h-incubation. **(C)** FCM histograms for different NPs in MCF-7 cells after 1 h-incubation. (*t*-test, ^∗^*P* < 0.05, ^∗∗^*P* < 0.01, ^∗∗∗^*P* < 0.001).

Since the endocytosis and sustained retention of NPs acts as a critical and final step on the therapeutic efficacy of NPs (Xu et al., 2012; Behzadi et al., 2017), the endocytosis of Apt-pD-CA-(PCL-*ran*-PLA) NPs was carefully investigated. We replaced the loaded DTX with a fluorescent molecule, coumarin-6 (C6), in order to study the endocytosis of all the developed NPs in MCF-7 cells. The CLSM images of MCF-7 cells after 2 h of incubation with 250 μg/mL of C6/CA-(PCL-*ran*-PLA) NPs, C6/pD-CA-(PCL-*ran*-PLA) NPs, and C6/Apt-pD-CA-(PCL-*ran*-PLA) NPs in DMEM are provided in **Figure 5A**. As could be observed from this figure, the fluorescence intensity (i.e., representing cellular uptake efficiency) within the MCF-7 cells did not show much difference between C6/CA-(PCL-*ran*-PLA) NPs and C6/pD-CA-(PCL-*ran*-PLA) NPs. Notably, the fluorescence intensity increased significantly within the MCF-7 cells incubated with C6/Apt-pD-CA-(PCL-*ran*-PLA) NPs, demonstrating the presence of AS1411 aptamers on NP surface may lead to high levels of *in vitro* cellular targeting efficacy.

In order to quantitatively verify the significant role of AS1411 aptamers during the endocytosis of NPs, MCF-7 cells were incubated with C6/CA-(PCL-*ran*-PLA) NPs, C6/pD-CA-(PCL-*ran*-PLA) NPs, and C6/Apt-pD-CA-(PCL-*ran*-PLA) NPs at the concentration of 100, 250, and 500 μg/mL, respectively. As shown in **Figure 5B**, the cellular uptake efficiency of C6/CA-(PCL-*ran*-PLA) NPs did not show much difference compared to that of C6/pD-CA-(PCL-*ran*-PLA) NPs at all concentrations. However, the cellular uptake efficiency of C6/Apt-pD-CA-(PCL-*ran*-PLA) NPs was 1.37-, 1.53-, and 1.91-fold of that of C6/CA-(PCL-*ran*-PLA) NPs at the concentration of 100, 250, and 500 μg/ml, respectively. In addition, similar results could also be confirmed by FCM assays (**Figure 5C**). Taken all together, the specific interactions between AS1411 aptamers and MCF-7 cells reinforcing the endocytosis of targeted C6/Apt-pD-CA-(PCL-*ran*-PLA) NPs compared to non-targeted C6/CA-(PCL-*ran*-PLA) NPs and C6/pD-CA-(PCL-*ran*-PLA).

### Effects of the Developed NPs on Cell Viability

**FIGURE 6:**
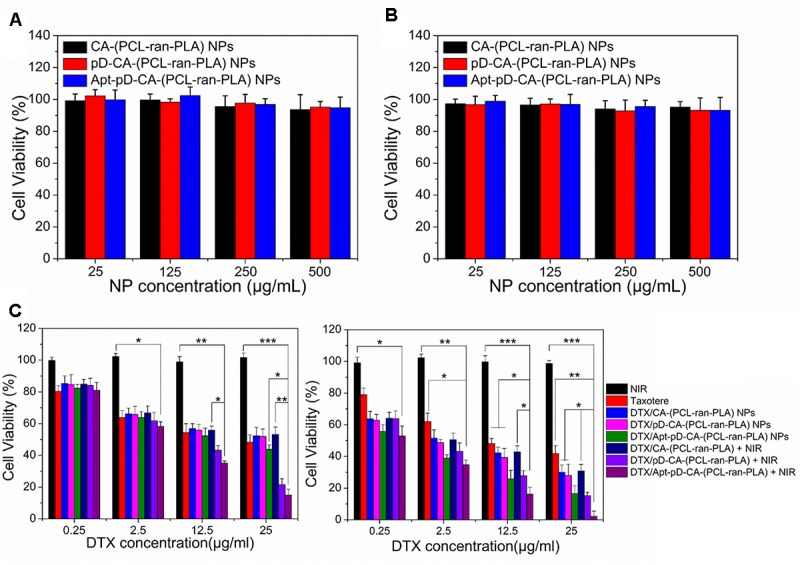
*In vitro* cytotoxicity of drug-free CA-(PCL-*ran*-PLA), drug-free pD-CA-(PCL-*ran*-PLA), and drug-free Apt-pD-CA-(PCL-*ran*-PLA) NPs after different incubation time: **(A)** 24 h, and **(B)** 48 h. **(C)** Viability of MCF-7 cells after various treatments at different DTX concentrations. The cells were cultured with DTX-loaded NPs in comparison with that of Taxotere^®^ at the same DTX dose for 24 h (left) and 48 h (right). NIR laser irradiation (808 nm, 10 min, 1.5 W/cm^2^) was performed immediately after adding different NPs before different incubation time (*t*-test, ^∗^*P* < 0.05, ^∗∗^*P* < 0.01, ^∗∗∗^*P* < 0.001).

MTT assays were carried out to evaluate the cytotoxicity of all the developed NPs. We first checked the *in vitro* toxicity of drug-free CA-(PCL-*ran*-PLA) NPs, pD-CA-(PCL-*ran*-PLA) NPs, and Apt-pD-CA-(PCL-*ran*-PLA) NPs at different concentrations (25, 125, 250, and 500 μg/mL) and after different incubation time (24 and 48 h). As shown in **Figures 6A,B**, none of these drug free NPs showed any functionalization of NPs (pD modification and aptamer conjugation) were biocompatible and non-toxic. Furthermore, the *in vitro* therapeutic efficacy of DTX/CA-(PCL-*ran*-PLA) NPs, DTX/pD-CA-(PCL-*ran*-PLA) NPs, and DTX/Apt-pD-CA-(PCL-*ran*-PLA) NPs without (i.e., chemotherapy groups) and with (i.e., chemo-photothermal therapy groups) the irradiation of NIR laser (808 nm, 10 min, 1.5 W/cm^2^) was performed in MCF-7 cells. Taxotere^®^, which is a clinical DTX formulation, was chosen as a reference. Group treated with only NIR laser were also chosen as a reference. MCF-7 cells were treated at 0.25, 2.5, 12.5, and 25 μg/mL of equivalent DTX concentrations for 24 and 48 h. For the chemo-photothermal therapy groups, NIR laser irradiation was performed immediately after adding different NPs before 24 or 48 h of incubation. It could be concluded from **Figure 6C**: (i) the cell viability decreased as the incubation time increased for both Taxotere^®^ and NP groups, indicating a time-dependent and dose-dependent effect; (ii) the DTX/Apt-pD-CA-(PCL-*ran*-PLA) NPs showed better *in vitro* therapeutic efficacy than Taxotere^®^, DTX/CA-(PCL-*ran*-PLA) NPs, DTX/pD-CA-(PCL-*ran*-PLA) NPs, and DTX/Apt-pD-CA-(PCL-*ran*-PLA) NPs. For instance, the cell viability of MCF-7 cells (48 h, at the DTX concentration of 12.5 μg/mL) was 48.2% for Taxotere^®^, 42.3% for DTX/CA-(PCL-*ran*-PLA) NPs, 39.4% for DTX/pD-CA-(PCL-*ran*-PLA) NPs, and 25.9% for DTX/Apt-pD-CA-(PCL-*ran*-PLA) NPs; (iii) the NIR laser irradiation did not change the viability of MCF-7 cells treated with DTX/CA-(PCL-*ran*-PLA) NPs. However, the cell viability significantly decreased for groups treated with DTX/pD-CA-(PCL-*ran*-PLA) NPs and DTX/Apt-pD-CA-(PCL-*ran*-PLA) NPs after the irradiation of NIR laser. For example, the cell viability of MCF-7 cells (48 h, at the DTX concentration of 12.5 μg/mL) was 42.8% for DTX/CA-(PCL-*ran*-PLA) NPs + NIR, 27.9% for DTX/pD-CA-(PCL-*ran*-PLA) NPs + NIR, and 16.2% for DTX/Apt-pD-CA-(PCL-*ran*-PLA) NPs + NIR. (iv) the DTX/CA-(PCL-*ran*-PLA) NPs and DTX/pD-CA-(PCL-*ran*-PLA) NPs showed similar viability at various concentrations, further demonstrating the surface coating of pD layers is biocompatible and non-toxic. In a word, AS1411 aptamers may have an effective targeting efficacy for NPs and the synergistic chemo-photothermal strategy showed the most significant therapeutic efficacy. Therefore, DTX/Apt-pD-CA-(PCL-*ran*-PLA) NPs could be employed as promising targeted drug delivery systems for effective chemo-photothermal cancer therapy.

### *In Vivo* Photothermal Imaging of DTX/Apt-pD-CA-(PCL-*ran*-PLA) NPs

**FIGURE 7:**
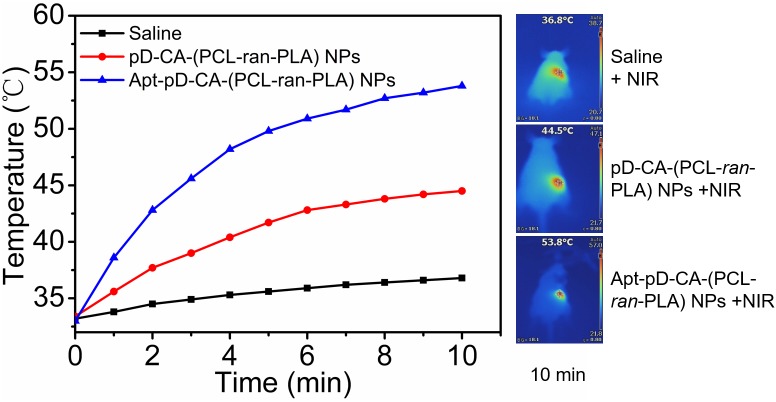
Time-dependent temperature changes of the tumor sites recorded by an IR camera under an 808 nm NIR laser (1.5 W/cm^2^). Saline, DTX/pD-CA-(PCL-*ran*-PLA) NPs and DTX/Apt-pD-CA-(PCL-*ran*-PLA) NPs were intravenous injected at the same volume and dose. After 12 h of injection, NIR laser irradiation was performed. Inset: Photothermal imaging after 10 min of irradiation.

Based on the promising *in vitro* targeting and therapeutic efficacy, we performed *in vivo* photothermal imaging of mice after intravenous (i.v.) injection of saline, DTX/pD-CA-(PCL-*ran*-PLA) NPs, and DTX/Apt-pD-CA-(PCL-*ran*-PLA) NPs respectively to further access the *in vivo* targeting efficacy. After 12 h of i.v. injection, NIR laser irradiation (808 nm, 10 min, 1.5 W/cm^2^) was performed at tumor sites. As shown in **Figure 7**, the temperature increments in the tumor sites of the mice treated with DTX/Apt-pD-CA-(PCL-*ran*-PLA) NPs and DTX/pD-CA-(PCL-*ran*-PLA) NPs were 20.8°C and 11.1°C respectively, reaching a temperature of 53.8°C and 44.5°C after irradiation. DTX/Apt-pD-CA-(PCL-*ran*-PLA) NPs group exhibited a higher temperature increment than DTX/pD-CA-(PCL-*ran*-PLA) NPs group, which could be explained by the active tumor targeting effect of AS1411 aptamers and higher accumulation of DTX/Apt-pD-CA-(PCL-*ran*-PLA) NPs in tumor sites. However, the tumor site temperature of mice received i.v. injection of saline did not show much increment with a highest temperature of 36.8°C, which was not enough for tumor ablation.

### *In Vivo* Antitumor Effects of NPs

**FIGURE 8:**
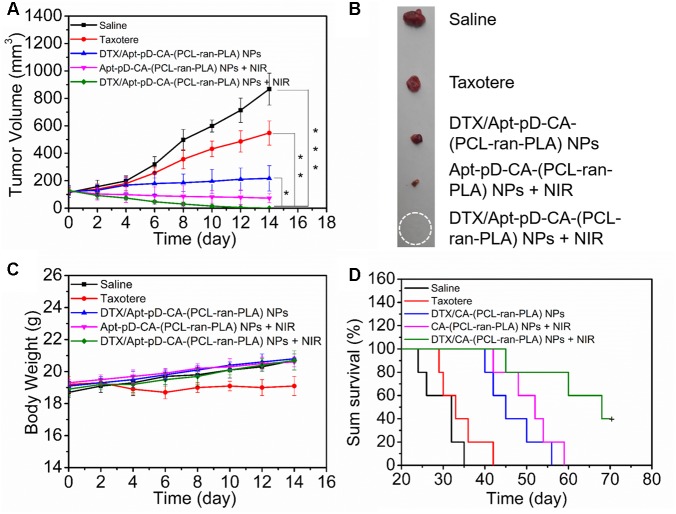
Anti-tumor efficacy of different treatments on the nude mice bearing MCF-7 xenograft. Taxotere^®^ and DTX/Apt-pD-CA-(PCL-*ran*-PLA) NPs were injected at the same DTX dose. After 12 h of injection, NIR laser irradiation was performed (808 nm, 10 min, 1.5 W/cm^2^). **(A)** Tumor growth curve after different treatments (*t*-test, ^∗^*P* < 0.05, ^∗∗^*P* < 0.01, ^∗∗∗^*P* < 0.001). **(B)** Images of tumors from each group at the end point of study. **(C)** Body weight curves of the nude mice during the treatment period. **(D)** Kaplan–Meier survival log-rank analysis of the nude mice bearing MCF-7 xenograft after different treatments.

The blood circulation time was firstly accessed by *in vivo* pharmacokinetic studies before *in vivo* antitumor studies. As shown in Supplementary Figure S1, all the prepared NPs have extended the blood circulation time of the DTX compared to Taxotere^®^, which may contribute to effective delivery of DTX *in vivo*. On the basis of the above *in vitro* cell experiments and *in vivo* targeting imaging studies, DTX/Apt-pD-CA-(PCL-*ran*-PLA) NPs were chosen to further verify the *in vivo* antitumor efficacy of this targeting chemo-photothermal strategy. As shown in **Figures 8A,B**, both commercial Taxotere^®^ and NP treatment groups could inhibit the tumor growth. In detail, all the NP treatment groups, i.e., DTX/Apt-pD-CA-(PCL-*ran*-PLA) NPs (target chemotherapy group), Apt-pD-CA-(PCL-*ran*-PLA) NPs + NIR (target photothermal group), and DTX/Apt-pD-CA-(PCL-*ran*-PLA) NPs + NIR (target chemo-photothermal therapy group), showed better *in vivo* antitumor efficacy than Taxotere^®^. The synergistic chemo-photothermal therapy group showed the highest therapeutic efficacy which significantly eliminated the tumors. These results were consistent with the above cellular experiments and *in vivo* targeting studies. Moreover, the body weight of the mice in all the NP treatment groups did not show any difference compared to the control group receiving saline, which that of Taxotere^®^ showed slightly weight loss, indicating the potential *in vivo* biocompatibility of DTX/Apt-pD-CA-(PCL-*ran*-PLA) NPs (**Figure 8C**). Another batch of nude mice were used to access the survival data of nude mice receiving different treatments (**Figure 8D**). As could be concluded from this figure, the survival time of “DTX/Apt-pD-CA-(PCL-*ran*-PLA) NPs + NIR” group was at least extended by 35, 28, 14, and 11 days compared to “Saline” group, “Taxotere^®^” group, “DTX/Apt-pD-CA-(PCL-*ran*-PLA) NPs” group, and “Apt-pD-CA-(PCL-*ran*-PLA) NPs + NIR” group. These results further demonstrated the significant therapeutic efficacy of this target chemo-photothermal therapy strategy based on DTX/Apt-pD-CA-(PCL-*ran*-PLA) NPs, indicating these NPs are able to be applied as promising targeting drug delivery systems for synergistic chemo-photothermal therapy of breast cancer.

## Conclusion

In this study, we for the first time reported the successful synthesis of robust DTX/Apt-pD-CA-(PCL-*ran*-PLA) NPs with star shaped CA-(PCL-*ran*-PLA) copolymers, which could be applied as promising targeting drug delivery systems for synergistic chemo-photothermal therapy of breast cancer. By surface modification with facile dopamine polymerization method, AS1411 aptamers were able to be simply conjugated on the surface of NPs for target delivery of drugs. The DTX/Apt-pD-CA-(PCL-*ran*-PLA) NPs were well characterized by surface morphology, LC and EE, stability, photothermal properties and drug release profiles. The *in vitro* and *in vivo* targeting effect of these NPs were also accessed. The *in vitro* cytotoxicity assays by MTT showed that DTX/Apt-pD-CA-(PCL-*ran*-PLA) NPs together with NIR laser irradiation (target chemo-photothermal therapy) could significantly inhibit cell proliferation compared with all other groups. The *in vivo* antitumor assays, as well as the improved survival time and reduced side effects, further confirmed the significant therapeutic effects of this target chemo-photothermal therapy strategy. All the results observed from the *in vivo* studies were consist with the *in vitro* assays, suggesting the robust DTX/Apt-pD-CA-(PCL-*ran*-PLA) NPs are promising in target delivery of drugs and synergistic chemo-photothermal therapy of breast cancer.

## Author Contributions

NK, MD, Y-DC, and X-BS conceived the idea and designed the study. NK and MD performed all the experiments and analyzed the data. X-NS helped in nanoparticle preparation and *in vitro* experimental assays. Y-DC and X-BS provided the technical support and corrections of the manuscript. NK wrote the manuscript and revised it according to the comments of Y-DC and X-BS.

## Conflict of Interest Statement

The authors declare that the research was conducted in the absence of any commercial or financial relationships that could be construed as a potential conflict of interest.
