# Supplementary material for: Polydopamine-Functionalized CA-(PCL-ran-PLA) Nanoparticles for Target Delivery of Docetaxel and Chemo-photothermal Therapy of Breast Cancer
Source: Front Pharmacol. 2018 Feb 21;9:125. doi: 10.3389/fphar.2018.00125 (PMC5829531; doi:10.3389/fphar.2018.00125)
Supplement: Supplementary file 1 [file Data_Sheet_1.DOC]

***Supplementary Information***

# Polydopamine-functionalized CA-(PCL-*ran*-PLA) nanoparticles for target delivery of docetaxel and chemo-photothermal therapy of breast cancer

Na Kong^1,2,3+^, Mei Deng^4+^, Xiu-Na Sun^1,2^, Yi-Ding Chen^4*^, Xin-Bing Sui^5,6,7*^

^1^ Sir Run Run Shaw Hospital, Zhejiang University School of Medicine, Hangzhou, Zhejiang, China

^2^ Institute of Translational Medicine, Zhejiang University, Hangzhou, Zhejiang, China

^3^ Center for Nanomedicine and Department of Anesthesiology, Brigham and Women’s Hospital, Harvard Medical School, Boston, Massachusetts, United States

^4^ Department of Surgical Oncology, The Second Affiliated Hospital, Zhejiang University School of Medicine, Hangzhou, Zhejiang, China

^5^Department of Medical Oncology, Holistic Integrative Oncology Institutes and Holistic Integrative Cancer Center of Traditional Chinese and Western Medicine, the Affiliated Hospital of Hangzhou Normal University, College of Medicine, Hangzhou Normal University, Hangzhou, Zhejiang, China

^6^Department of Cancer Pharmacology, Holistic Integrative Pharmacy Institutes, College of Medicine, Hangzhou Normal University, Hangzhou, Zhejiang, China

^7^Key Laboratory of Elemene Class Anti-cancer Chinese Medicine of Zhejiang Province and Engineering Laboratory of Development and Application of Traditional Chinese Medicine from Zhejiang Province, Hangzhou Normal University, Hangzhou, Zhejiang, China

* Correspondence:

[ydchen@zju.edu.cn](mailto:ydchen@zju.edu.cn) (Y-D.C.); [hzzju@zju.edu.cn](mailto:hzzju@zju.edu.cn) (X-B.S.)

**Pharmacokinetic studies**

All the protocols for animal experiments were approved by the Administrative Committee on Animal Research in the Zhejiang University. Male Sprague-Dawley (SD) rats of 200 ± 10 g were obatined for the *in vivo* pharmacokinetic studies. The mice were acclimatized at temperature of 25 °C under natural light/dark conditions for 7 days before experiments. After that, the SD rats were randomly divided into four groups (n = 5) and treated with Taxotere®, DTX/CA-(PCL-*ran*-PLA) NPs, DTX/pD-CA-(PCL-*ran*-PLA) NPs, and DTX/Apt-pD-CA-(PCL-*ran*-PLA) NPs (DTX dose of 10 mg/kg) *via i.v.* injection, respectively. Blood samples of all groups were collected from the rim of eyes at 5 min, 15 min, 30 min, 1 h, 2 h, 4 h, 8 h, 12 h and 24 h after *i.v.* administration. Plasma samples were harvested by centrifugation (4,000 rpm, 10 min) and stored at -20 °C for HPLC analysis. DTX in the plasma samples was extracted into 1 ml of diethyl ether and was allowed to evaporate in separate tubes. 100 ml of HPLC mobile phase A (Vacetonitrile : Vmethanol : Vwater = 45: 5: 50) was added to the dried tubes and centrifuged at 12,000 rpm for 15 min. 90 ml of the supernatant was transferred to HPLC vial inserts and 50 ml was injected into the column. By increasing the proportion of mobile phase A from 0 % to 100% in 50 min, an elution gradient was applied. The system was then brought to initial condition of 100% mobile phase B (Vacetonitrile : Vmethanol : Vwater = 40: 5: 50) and equilibrated for 4 min by holding at 100% mobile phase B. The flow rate was kept at 1 ml/min and the total run time was 55 min. Through the standard curve obtained for known concentrations of DTX in plasma, the DTX concentrations in plasma of all groups were determined. All the results were dose-normalized and plotted as plasma DTX concentration-time curves.

**Figure S1.** Drug concentration-time profile following intravenous administration of Taxotere®, DTX/CA-(PCL-*ran*-PLA) NPs, DTX/pD-CA-(PCL-*ran*-PLA) NPs, and DTX/Apt-pD-CA-(PCL-*ran*-PLA) NPs in SD rats at the DTX dose of 10 mg/kg (n = 5).
